# Metagenomic analysis of viral diversity in Portuguese bats

**DOI:** 10.1007/s11259-025-10888-5

**Published:** 2025-09-20

**Authors:** Mahima Hemnani, Mustafa Karatas, Andreia V S Cruz, Priscilla Gomes da Silva, Gertrude Thompson, Patrícia Poeta, Hugo Rebelo, Jelle Matthijnssens, João R Mesquita

**Affiliations:** 1 School of Medicine and Biomedical Sciences, Porto University, Porto, 4050-313 Portugal; 2 Department of Microbiology, Immunology, and Transplantation, Rega Institute, Laboratory of Viral Metagenomics, KU Leuven, Leuven, Belgium; 3 Department of Protein Science, Affinity Proteomics Division, Science for Life Laboratory (SciLifeLab), KTH Royal Institute of Technology, Stockholm, Sweden; 4 Research Centre in Biodiversity and Genetic Resources, CIBIO-InBIO, University of Porto, Campus de Vairão, Vairão, 4485-661 Portugal; 5 Microbiology and Antibiotic Resistance Team (MicroART), Department of Veterinary Sciences, University of Trás-os-Montes and Alto Douro (UTAD), Vila Real, 5000-801 Portugal; 6 Associated Laboratory for Green Chemistry (LAQV), Chemistry Department, Faculty of Science and Technology, University NOVA of Lisbon, Caparica, 2829-516 Portugal; 7 Associate Laboratory for Animal and Veterinary Science (AL4AnimalS), University of Trás- os-Montes and Alto Douro (UTAD), Vila Real, 5000-801 Portugal; 8 Veterinary and Animal Research Centre (CECAV), University of Trás-os-Montes and Alto Douro (UTAD), Vila Real, 5000-801 Portugal; 9 cE3c—Centre for Ecology, Evolution and Environmental Changes & CHANGE—Global Change and Sustainability Institute, Departamento de Biologia Animal, Faculdade de Ciências, Universidade de Lisboa, Lisboa, Portugal; 10 Centro de Estudos de Ciência Animal (CECA), Instituto de Ciências, Tecnologias e Agroambiente (ICETA), Universidade do Porto (UP), Rua D. Manuel II, Apartado, Porto, 55142, 4051-401 Portugal; 11 Associate Laboratory for Animal and Veterinary Science (AL4AnimalS), Lisboa, 1300-477 Portugal

**Keywords:** Bat, Coronavirus, Picornavirus, Adenovirus, Dependoparvovirus, Metagenomics

## Abstract

Bats are highly diverse mammals and known reservoirs of numerous zoonotic viruses. Their role in the ecology of emerging infectious diseases continues to be of significant interest. This study aimed to evaluate the occurrence of coronaviruses (CoVs) in Portuguese bats and predict the affinity of their spike proteins with the aminopeptidase N (APN) receptor of several host species. The study also explored the viral diversity in bat samples using metagenomic sequencing. Ten bats (five *Myotis myotis* and five *Miniopterus schreibersii*) were captured at an underground roost in 2022 (Central Portugal), and fecal samples, oral, and anal swabs were collected (*n* = 27). A Pan-CoV nested RT-PCR was used for initial screening, followed by viral metagenomic sequencing of all fecal samples and one CoV-positive buccal swab. In silico protein docking studies were performed between a Portuguese bat CoV spike protein and APNs of bats, pigs, and humans. Pan-CoV nested RT-PCR identified three positive samples: two fecal samples and one buccal sample. Metagenomic sequencing allowed us to determine two near complete CoV genomes. Protein docking predicted strong binding of this spike protein to bat, porcine, and human APN receptors. Metagenomics also identified picornaviruses, adenovirus, and dependoparvovirus in fecal samples. This study reports the first near complete genome sequences of two members of the *Alphacoronavirus* genus from a Portuguese bat The identification of other viral families highlights the diverse virome of these cave-dwelling bat species. Protein docking studies suggest a potential for cross-species transmission of this bat CoV between bats, porcines and humans, though further research is needed to confirm these interactions.

**Supplementary Information:**

The online version contains supplementary material available at 10.1007/s11259-025-10888-5.

## Introduction

Bats, belonging to the order Chiroptera, are a highly diverse taxa with over 1,400 species identified worldwide (Bokelmann and Balkema-buschmann 2021; Wang et al. 2023), and of great relevance in a One Health context (Figueiroa et al. 2025). They are the only mammals capable of sustained flight and have successfully adapted to a wide range of ecological niches, enabling them to inhabit a vast array of habitats and roosts (Irving et al. 2021; Soler-Tovar and Escobar 2025).

These animals represent a unique group of mammals that have evolved to coexist and be a reservoir for a vast diversity of viruses (Tiwari et al. 2020; Gupta et al. 2021), such as *Coronaviridae*, *Adenoviridae*, *Parvoviridae*, *Picornaviridae*, *Rhabdoviridae*, *Herpesviridae*, *Filoviridae*, *Paramyxoviridae*, *Astroviridae* (Calisher et al. 2006; Drexler et al. 2012; Smith and Wang 2013; Moreno et al. 2017; Wang and Anderson 2019; Gupta et al. 2021) and members of a total of 66 genera from 15 families of RNA and five families of DNA viruses have been detected from 75 bat genera. Over millions of years, these animals have developed a specialized immune system that allows them to harbor these viruses (Apoorva and Singh 2024), several of which have zoonotic potential (Irving et al. 2021; Soler-Tovar and Escobar 2025; Pujol et al. 2025).

They are also natural reservoirs for coronaviruses (CoVs) related to those that have caused outbreaks in humans, such as SARS, MERS and SARS-CoV-2 (El-Sayed and Kamel 2021), even though these specific human pathogens have not been directly detected from bats. On the other hand, their unique ability to fly enables them to migrate over long distances, significantly increasing the potential for both inter- and intra-species transmission (Drexler et al. 2014; Gupta et al. 2021). A recent systematic review reported that, to date, there are 30 studies in Europe that have examined the presence of CoVs in bats, covering countries such as Italy, Germany, the Netherlands, Ukraine, Belgium, Bulgaria, Denmark, Finland, France, Hungary, Luxembourg, Romania, Slovenia, Spain, Sweden, Switzerland, Poland, the United Kingdom, and Portugal (Hemnani et al. 2024). Bats are known to host at least 30 different CoVs with complete genome sequences available, representing different species or lineages, and many more when including those lacking full genomes (Platto et al. 2021).

CoVs possess a non-segmented, positive-stranded RNA genome, with a typical size of 27–32 kb, making it the largest among all RNA viruses (Nakagawa and Miyazawa 2020). Their virions are enveloped, having structures protruding from the surface called “spikes” (Domańska-Blicharz et al. 2021). Their lengthy genomes, high rates of recombination, and frequent mutations (Woo et al. 2009; Grellet et al. 2022) can facilitate the infection of a wide range of hosts, including mammals, birds, amphibians, and teleost fish (Miller et al. 2021), and adapt to new host species with modified pathogenicity (Drzewnioková et al. 2021). This adaptability results in diverse clinical outcomes, from asymptomatic cases to severe and sometimes fatal diseases (Woo et al. 2023). CoVs are classified into four genera: *Alphacoronavirus* (AlphaCoV) and *Betacoronavirus* (BetaCoV), which primarily cause diseases in mammals and are considered pathogenic, and *Gammacoronavirus* (GammaCoV) and Deltacoronavirus (DeltaCoV), which evolved from CoVs originally found in birds. While most GammaCoV and DeltaCoV strains cause diseases in birds, this is not exclusively the case (Mihindukulasuriya et al. 2008).

Recent efforts have increasingly focused on using metagenomic techniques (Kim et al. 2024) to explore the vast array of pathogens present in wildlife species (Prentice et al. 2024). Considering the extensive diversity of viruses and the rising frequency of direct and indirect interactions at the human-domestic and animal-wildlife interface (Cui et al. 2023), it is likely that many viruses remain unidentified, along with numerous unrecognized instances of cross-species transmission. Advancing our knowledge of the viruses involved in spillover events and successful host transitions is essential for deepening our understanding of the factors driving the emergence of new diseases (Ruiz-Aravena et al. 2022; Gangopadhayya and Bhukya 2023).

Usually, studying viromes can be challenging because most of the genetic material in a sample is of non-viral origin (Reyes et al. 2012). Next-generation sequencing has transformed the discovery of new viruses in humans and animals across diverse ecosystems (Bodewes et al. 2014), especially with new approaches that include enrichment for virus-like-particles (VLPs) from a sample and the performance of random amplification, if the starting material needs to be increased before NGS library preparation (Reyes et al. 2012; Conceição-Neto et al. 2015a).

Molecular docking is a widely used tool to simulate the 3D structure of protein-ligand or protein-protein complexes, examining their interactions, and predicting binding affinities (Andrusier et al. 2008). The CoV spike protein is a transmembrane homotrimeric protein required for attachment to the host cell-specific receptors and subsequent catalysis of the virus-host cell membrane fusion (Guruprasad 2021). In CoVs, it extends from the viral surface, giving them their characteristic crown-like appearance (Ghosh et al. 2021). One such receptor is aminopeptidase N (APN), a membrane-bound metalloprotease involved in peptide digestion, which also functions as a viral receptor for certain coronaviruses, facilitating entry into host cells (Reguera et al. 2012).

Despite increasing awareness of bats as natural reservoirs for diverse viruses, including coronaviruses (CoVs), there is still a lack of data on CoV circulation in European bat populations, particularly in regions like Portugal where surveillance has been limited. Moreover, the potential for cross-species transmission through molecular mechanisms such as receptor binding remains poorly explored in these populations.

Therefore, the aim of this study is to investigate the occurrence of CoVs in bats from an underground roost in Portugal, while also utilizing viral metagenomics to explore the CoV genome as well as other potential viral families co-circulating within these hosts. Computational protein docking studies were also employed to predict the binding affinity between the spike protein of bat CoV and the APN receptor of mammal species like bats, porcines and humans to predict infection. This research underscores the importance of a One Health approach to understanding the interconnectedness of human, animal, and environmental health, which is crucial for preventing zoonotic outbreaks and promoting public health resilience.

## Materials and methods

### Bat sampling

Bats were captured in July 2022 at a Montemor I underground roost (municipality of Montemor-o-Novo, Central Portugal), a shelter for the conservation of species such as the greater mouse-eared bat (*Myotis myotis*) and the common bent-wing bat (*Miniopterus schreibersii).*

We used hand nets for bat capture, resulting in a total of ten individuals from two species: *M. myotis* (*n* = 5) and *M. schreibersii* (*n* = 5). Each bat was carefully Handled to prioritize its well-being, with species identification conducted using standard morphological techniques. After capture, the bats were placed in individual cotton bags for 5 to 20 min, during which time three types of matrices were collected namely fresh fecal samples (*n* = 7), anal (*n* = 10) and buccal swabs (*n* = 10). Each swabbed matrix was placed in an individual sterile Eppendorf tube and stored dry at *-*20 °C until further processing, without the addition of buffer. To minimize stress and disruption, after processing, the bats were safely released and promptly returned to their natural environment, enabling them to quickly resume normal behavior with minimal interference. This approach not only ensured the bats’ well-being but also preserved the ecological integrity of their habitat. All capture and handling procedures were carried out in full compliance with permits issued by the Instituto da Conservação da Natureza e das Florestas (ICNF), adhering strictly to the regulations and guidelines set forth by the conservation authority (license number: 274/2023/CAPT).

### Screening for CoVs

All samples were immediately stored at *-*20 °C until further processing. During preparation for PCR, they were vortexed in 500 µL of PBS and RNA was extracted using the QIAamp viral mini kit (QIAGEN, Hilden, Germany). The RNA was stored at − 80 °C and later tested for CoVs using a pan-CoV nested RT-PCR (Drzewnioková et al. 2021) assay targeting a conserved region of the RNA-dependent RNA polymerase (*RdRp*) gene, resulting in a final product measuring 440 bp. The sensitivity of the primers used was previously validated for broad-spectrum CoV detection and it has been documented that utilizing a small segment of the *RdRp* from CoVs is an approach for determining taxonomic classifications even at the subgenus level (Drzewnioková et al. 2021; Wilkinson et al. 2021). Amplicons of the expected size were purified using the GRS PCR Purification Kit (GRiSP, Porto, Portugal). Bidirectional sequencing was then performed using the target gene’s specific primers by the Sanger method. Sequence alignment was performed using the BioEdit Sequence Alignment Editor v7.1.9 software package, version 2.1 (Ibis Biosciences, Carlsbad, CA, USA). The obtained sequences were trimmed, and consensus sequences were compared with sequences available in the GenBank database (https://www.ncbi.nlm.nih.gov/).

### Sample preparation and metagenomic sequencing

All seven fecal samples and the one buccal swab sample that tested positive for CoVs by the pan-CoV nested RT-PCR were further analyzed by NGS. For viral metagenomics sample preparation the NetoVIR (Novel enrichment technique of VIRomes) protocol was used as previously described (Conceição-Neto et al. 2015b, 2017; Yinda et al. 2017). 

Subsequently, fecal suspensions and the buccal sample were prepared at a concentration of 10% (w/v) in sterile PBS and homogenized at 3,000 rpm for 1 min using a tissue homogenizer (Precellys, France). The homogenized samples were centrifuged at 17,000 × g for 3 min, and the supernatant containing VLPs was collected. This supernatant was filtered through 0.8 μm PES filters (Sartorius) to remove larger debris. The filtered supernatant was treated with a nuclease cocktail consisting of Benzonase (Novagen) and Micrococcal Nuclease (New England Biolabs) at 37 °C for 2 h to degrade free-floating nucleic acids. After the nuclease treatment, viral RNA and DNA were extracted using the QIAamp Viral RNA Mini Kit (QIAGEN), following the manufacturer’s protocol with a modification to exclude carrier RNA from the lysis buffer.

For the preparation of sequencing libraries, random amplification was performed using the Whole Transcriptome Amplification (WTA2) Kit (Sigma-Aldrich). Amplification included first and second strand synthesis followed by PCR, which was conducted over 17 cycles with a denaturation temperature set at 95 °C to ensure complete denaturation of both dsDNA and dsRNA. The amplified products were then purified using MSB Spin PCRapace spin columns (Stratec).

For library preparation, we followed the Nextera XT Library Preparation Kit protocol (Illumina). The DNA from the WTA2 amplification was tagmented by adding 7.5 µL of tagmentation mix to each sample in a 96-well plate, followed by the addition of 2.5 µL of the DNA sample. After a brief spin and incubation in a PCR machine, 2.5 µL of Neutralize Tagment Buffer was added. The samples were then indexed by adding 5 µL of unique indexes from a 96-well plate, followed by the addition of 7.5 µL of Nextera PCR Master Mix. After PCR amplification, the products were purified using AMPure XP beads at a 0.6× bead-to-DNA ratio. This included two ethanol washes and resuspension in 26.25 µL of Tris-HCl (Tris(hydroxymethyl)aminomethane hydrochloride) buffer. Finally, the prepared libraries were sequenced on an Illumina HiSeq 2500 platform, generating 2 × 150 bp paired-end reads across 300 cycles.

### Bioinformatic analysis of metagenomic sequencing

On average, 35 M reads per sample were obtained. Raw sequencing reads were processed using the ViPER pipeline (De Coninck et al. 2025). Shortly, low quality reads and adapters were trimmed by Trimmomatic (Bolger et al. 2014) (v.039) and remaining reads were *de novo* assembled with SPAdes (v3.15.3) into contigs (Prjibelski et al. 2020).

The assembled contigs were taxonomically assigned using DIAMOND v.2.0.9, employing the NCBI non-redundant (nr) protein database (accessed on 17 March 2023) for sequence alignment. A lowest common ancestor (LCA) approach was utilized to assign taxonomy, which was facilitated by TaxonKit v.0.8.0. Additionally, KronaTools v.2.8 was employed to visualize the taxonomic composition of the viral communities. Contigs were classified into eukaryotic viruses, phages, bacteria and eukaryotes. We focused exclusively on viruses capable of infecting bats, excluding those infecting archaea, bacteria, fungi, invertebrates, and plants from the analysis.

In parallel, EsViritu (Tisza et al. 2023) was applied to further refine the taxonomic resolution as a reference-guided assembly approach, particularly focusing on the detection and classification of well represented human and animal viruses in existing databases. EsViritu’s integration with the *de novo* assembly approach provided additional confidence in the classification of the viral contigs.

The genomic organization of the near-complete genomes was assessed using the Genome Detective Virus Tool (https://www.genomedetective.com/app/typingtool/virus/). This tool facilitated annotation of open reading frames (ORFs) and detection of conserved genomic features. Detailed genome characterizations, including ORF structure and genome lengths, are available in the Supplementary File.

### PCR primers designed for full Spike characterization

In one of the Cov sequences (MiSc-F47-CoV-2), a small region of the spike gene was not recovered through NGS. To obtain the missing region of the spike gene, specific primers were designed (Table 1) for targeted amplification and sequencing using the Sanger method.

**Table 1:** Primers designed for the amplification and sequencing of the missing Spike gene region using Sanger sequencing

Primer	Sequence (5’ → 3’)
Forward	5- TGG CAC CAT TGG TAA AGG CT −3
Reverse	5- AGG TCA AGC GCT GTT TCA GA −3

We employed the one-step RT-PCR kit (GRiSP^®^, Porto, Portugal) for the initial round of PCR. The following conditions were used in the Veriti 96-well thermal cycler (Thermo Fisher) for amplification reactions: an initial cycle of 3 min at 95 °C, followed by 40 cycles of 62 °C, then 67 °C, and then temperatures continuing to drop in subsequent cycles to levels to avoid spurious amplification, 50 °C for 15 s, and 72 °C for 2 s, with a final elongation at 72 °C for 10 min.

### Phylogenetic analysis

Phylogenetic trees were constructed using the assembled genome of CoVs, picornaviruses and adenovirus. Reference strains of these viral families with a query coverage exceeding 70% were retrieved from the NCBI database using BLASTn (Chen et al. 2015).

After removing duplicate genome sequences with BBMap (Smith and Yun 2017), alignments were generated using MAFFT (Gu et al. 2025), trimmed with Trimal (Capella-Gutiérrez et al. 2009), and maximum likelihood phylogenetic trees were constructed with IQ-TREE 2 (Minh et al. 2020).

### Nucleotide sequences accession numbers

The viruses reported in this article are available in the GenBank database (OQ613362, PV221239, OQ613361, PV359018, PV359019, PV383542-PV383552). The relevant raw high throughput sequencing data obtained in this study were deposited at the NCBI Sequence Read Archives (SRA) under BioProject ID PRJNA1162723.

### Homology modelling and protein-protein Docking simulations

Protein docking simulations were performed to assess the binding affinity between the F47 CoV spike protein obtained in this study, and bat, porcine and human APNs. The crystal structures of the porcine epidemic diarrhea virus (PEDV) spike protein, as well as human and porcine APNs were obtained from the Protein Data Bank (PDB) (Berman et al. 2000) (http://www.rcsb.org/, accessed on October 15, 2024). The PDB IDs for these structures are 6U7K, 6U7G, and 5LDS, respectively.

In the absence of structure models for bat APN, a 3D model was generated via homology modeling using the SWISS-MODEL platform (Waterhouse et al. 2018) (https://swissmodel.expasy.org/), with porcine APN (PDB ID: 5LG6) serving as the template. For this purpose, the amino acid sequence of APN from *Miniopterus natalensis* (XP_016072651) was retrieved from GenBank (https://www.ncbi.nlm.nih.gov/genbank/, accessed on October 30, 2024).

The 3D model of the CoV spike protein obtained in this study through full genome sequencing was built through homology modelling using the SWISS-MODEL platform, with the crystal structure of PEDV spike (PDB ID: 6U7K) as the template.

The spike proteins and bat APN were preprocessed using the Chimera Minimize Structure tool (version 1.18) and saved in PDB format. For the human and porcine APN proteins, preparation was carried out using Chimera’s Dock Prep tool (version 1.18), and these structures were also saved in PDB format.

Protein docking studies were conducted to evaluate the risk of cross-species transmission because the spike protein’s receptor-binding domain (RBD) determines host specificity by binding to host cell receptors. Protein-protein docking studies between spike RBD and APN proteins were performed in triplicate using Haddock 2.4 (Honorato et al. 2021, 2024), with default parameters. Active residues were specified to provide insights into interacting residues (Deng et al. 2016; Ji et al. 2022; Xu et al. 2023), while passive residues were automatically assigned based on the active residues, including all residues on the interface surface within a 6.5 Å radius of any active residue. The cluster models with the highest HADDOCK scores were saved in PDB format.

## RESULTS

### Screening for CoVs

In this study, samples were collected from ten bats, including ten buccal swabs, ten anal swabs, and seven fecal samples. Using pan-CoV nested RT-PCR on all 27 samples, three samples (7.41%; 95% confidence interval [CI]: 0% to 17.29) yielded amplicons of the expected size for coronavirus (440 bp), which were subsequently Sanger sequenced. This 440 bp fragment corresponds to a conserved region of the RNA-dependent RNA polymerase (RdRp) gene, chosen because of its high conservation across coronaviruses, enabling reliable comparison among diverse viral strains. Additionally, this region has been widely used in previous studies for coronavirus classification and evolutionary analyses, providing a robust phylogenetic signal for reconstructing evolutionary relationships within the group. Two sequences were retrieved from a fecal sample (MyMy-F52-CoV and, MyMy-F47-CoV), and one from a buccal swab (MyMy-B51-CoV) (Table 2).

**Table 2:** Sample details, including host species, sample type, identified virus, and proposed virus nomenclature for alphacoronaviruses detected in *M. myotis* and *M. schreibersii* samples through PCR-based screening

Sample ID	Species	Sample Type	Virus Identified	Proposed Virus Name
F47	*M. schreibersii*	Fecal	*Alphacoronavirus*	MiSc-F47-CoV
F52	*M. myotis*	Fecal	*Alphacoronavirus*	MyMy-F52-CoV
B51	*M. myotis*	Buccal swabs	*Alphacoronaviru*s	MyMy-B51-CoV

BLASTn revealed that sequence F52 was most closely related to several CoV strains from various bat species, including *Miniopterus schreibersii* from Portugal (OQ613364, Bat coronavirus isolate AN25), *Rhinolophus ferrumequinum* from Portugal (OQ613363.1, Bat coronavirus isolate F30), and *Miniopterus schreibersii* from Bulgaria (GU190240.1/BR98-55/BGR/2008), with percentage sequence identities ranging from 95 to 98%. Sequence B51 showed the closest matches to *Miniopterus schreibersii* from Portugal (OQ613364, Bat coronavirus isolate AN25) and Bulgaria (GU190240.1/BR98-55/BGR/2008), with sequence identities between 95% and 98%. Sequence F47 was most closely related to a *Miniopterus schreibersii* from Lebanon (MW880977.1, coronavirus isolate Alpha-CoV/LBN/LB20-CO-BAT-51 A/2020), bat guano from a mixed colony in Croatia (MZ558551.1 Bat coronavirus isolate BD8 ORF1ab polyprotein), *Miniopterus schreibersii* from France (KY423482.1, Alphacoronavirus sp. isolate FRA_EPI8_Misch35_8D_P11) and *Myotis punicus* from Algeria (MN701038.1, Alphacoronavirus sp. isolate 11 F), with sequence identities around 98%.

### Metagenomic sequencing of bat fecal samples

Previous studies from our team have shown that fecal samples contain the most viruses, suggesting that replication predominantly occurs in the gastrointestinal tract and emphasizing the potential for fecal-oral transmission (Hemnani et al. 2023). Based on this, we prioritized the 7 fecal samples for metagenomic analysis, along with the only buccal swab sample that tested positive by RT-PCR for CoV. Contigs were classified into eukaryotic viruses, phages, bacteria and eukaryotes, and we focused only on eukaryotic viruses. We detected viruses belonging to the following families: *Coronaviridae*, *Picornaviridae*, *Adenoviridae* (genus *Mastadenovirus*) and *Parvoviridae* (genus *Dependoparvoviridae)* (Table 3). Notably, all virus-positive samples resulting from the metagenomic sequencing were fecal, and no viral reads were detected in the buccal swab sample despite its RT-PCR positivity for CoV.

**Table 3:** Summary of viral sequences detected in *M. myotis* and *M. schreibersii* fecal samples through NGS, including viral classification, proposed nomenclature, and sequence lengths

Sample ID	Species	Sample Type	Virus Identified	Proposed Virus Name	Sequence length (nt)
F45	*M. myotis*	Fecal	Picornavirus	MyMy-F45-PicoV-1	7,421
			Parvovirus (*Dependoparvovirus*)	MyMy-F45-DepPV-1	2,359
				MyMy-F45-DepPV-2	1,120
				MyMy-F45-DepPV-3	3,138
				MyMy-F45-DepPV-4	1,128
			Coronavirus (*Alphacoronavirus*)	MyMy-F45-CoV-1	27,692
			Adenovirus (*Mastadenoviru*s)	MyMy-F45-Mast-1	36,906
F47	*M. schreibersii*	Fecal	Coronavirus (*Alphacoronavirus*)	MiSc-F47-CoV-2	28,712
F49	*M. schreibersii*	Fecal	Picornavirus	MiSc-F49-PicoV-2	8,557
F50	*M. myotis*	Fecal	Picornavirus	MyMy-F50.1-PicoV-3	8,351
				MyMy-F50.2-PicoV-3	7,656
F51	*M. myotis*	Fecal	Picornavirus	MyMy-F51.1-PicoV-4	9,293
				MyMy-F51.2-PicoV-4	8,440

### Two near complete genomes of CoVs were identified

Two near complete CoV genomes were assembled (MyMy-F45-CoV-1 and MiSc-F47-CoV-2). BLASTn revealed that sample F45 was most closely related to several CoV strains from various bat species, including *M. petax* from South Korea (ON378804. Alphacoronavirus sp. isolate BatCoV_B20-104-1), *M. adversus* and *M. chinensis* from China (OQ175102 Bat CoV MaJX20 isolate BtMa-AlphaCoV/JX2020-Q82 and MZ328298.1 Jingmen Myotis chinensis alphacoronavirus 1), and *M. dasycneme* from Denmark (MN535734.1 Bat coronavirus isolate BtCoV/18802-1/M.das/DK/2016). The percentage sequence identity between F45 and these related CoV sequences ranged from 81.3 to 87.2%.

Sample 47 (F47 MiSc-F47-CoV-2) collected from *M. schreibersii* also tested positive through PCR. A small region of the spike protein was missing. Specific primers were designed to amplify and characterize this missing region. This sample was most closely related to CoVs previously detected in *M. fuliginosus* and *M. schreibersii* from China (KJ473798.1 BtMf-AlphaCoV/HuB2013 and OQ175329.1 Bat Coronavirus MsYN20 isolate BtMs-AlphaCoV/YN2020-Q81), with percentage sequence identities ranging from 92.8 to 98.0%.

**Fig. 1:**
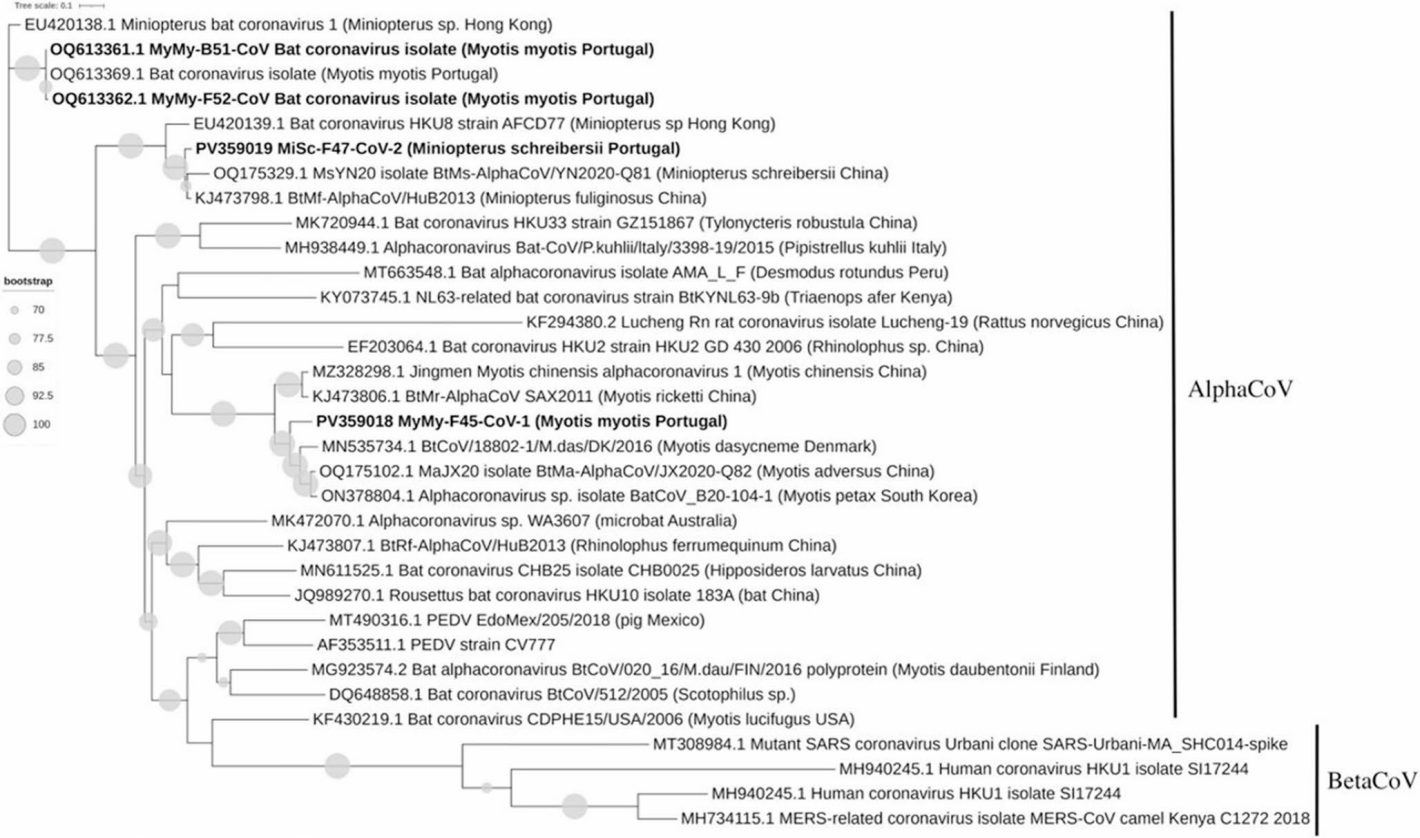
Phylogeny of the selected CoV strains based on the 440 bp partial RdRp gene. The tree was constructed for AlphaCoV, with BetaCoV as an outgroup. We used 30 reference strains, and the four CoV sequences identified in this study via metagenomics and PCR are indicated in bold. Sequences were aligned with MAFFT, and the phylogenetic tree was inferred using IQ-TREE2 with the maximum likelihood method based on the GTR + G model, with 1000 bootstrap replicates

Phylogenetic analysis confirmed that both samples belong to the AlphaCoV genus based on a 440 bp region of the RdRp gene (Fig. 1). This gene provides valuable taxonomic information and is highly effective for classification down to the subgenus level (Wilkinson et al. 2021). To better represent all CoV-positive samples, we generated an additional phylogenetic tree based on a 440 bp fragment of the RdRp gene, which includes all four samples testing positive by either RT-PCR or NGS.

**Fig. 2:**
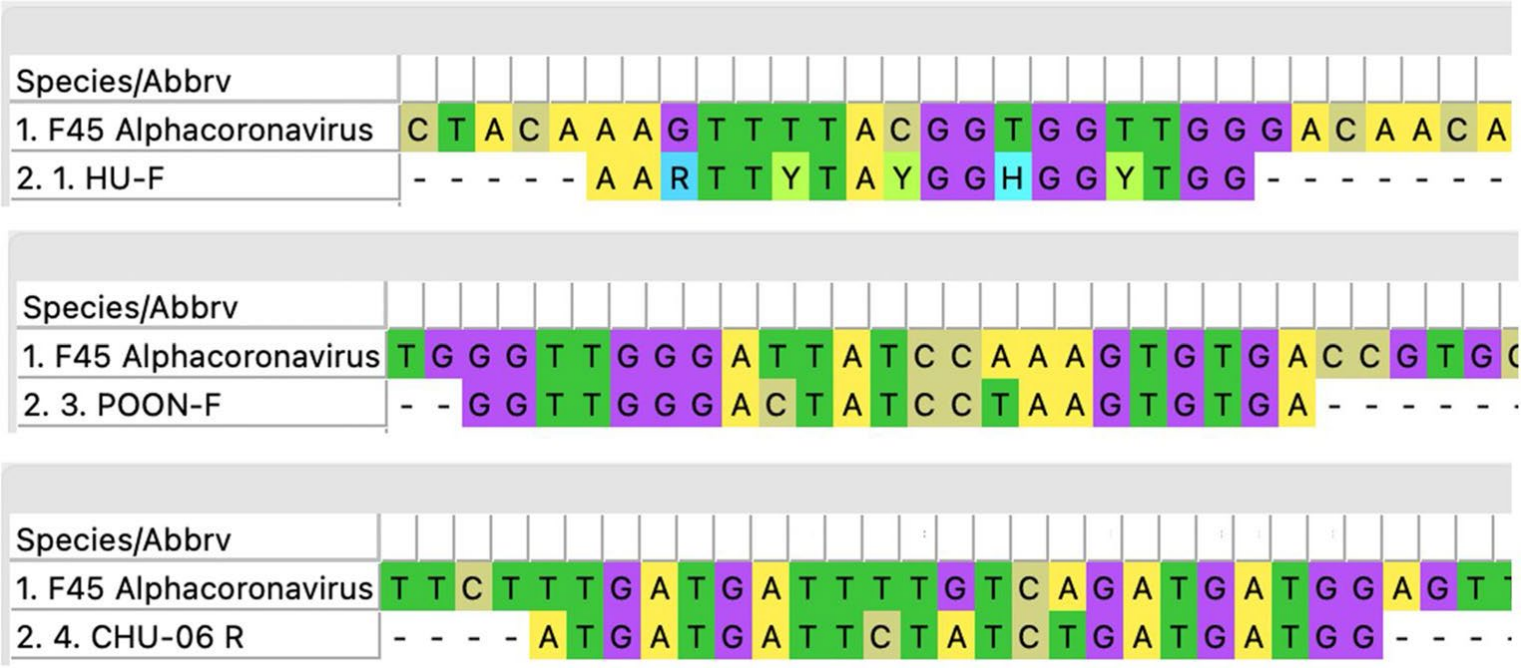
Alignment of primers with the F45 sequence. The Hu-F primer aligns perfectly. Chu-06 and Poon-F primers display four and two mismatches, respectively. The mismatches and ambiguous bases may have impaired primer annealing during PCR, contributing to the discrepancy between PCR and NGS results

The F45 sample initially tested negative for CoV by PCR was detected as positive through NGS analysis. Primer alignment with the F45 genome revealed complete alignment with HU-F primer and mismatches with both Chu-06 and Poon-F primers. A gap in the genome in the region of the HU-R primer hampered alignment analysis (Fig. 2).

### Identification of six highly divergent picornaviruses

**Fig. 3:**
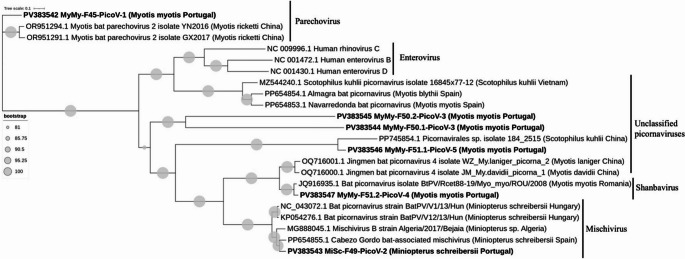
Phylogeny of the Picornaviruses found in this study. The tree was constructed based on the whole genome nucleotide sequences and using 16 reference strains. The tree was constructed using IQ-TREE2 with the maximum likelihood method based on the GTR + F model, including 1000 bootstrap replicates. Sequences were aligned with MAFFT. Sequences from this study are indicated in bold

We detected six near complete picornavirus genomes in four samples (F45, F49, F50, and F51). BLASTn analysis based on nucleotide sequences revealed varying levels of sequence divergence: while some genomes exhibited high divergence from known genomes (< 80% identity), others showed high similarity (up to 97.9% identity). Figure 3 shows the phylogenetic relationships among the picornaviruses identified in this study based on their whole genome nucleotide sequences. Sample F45 (MyMy-F45-PicoV-1), also positive for CoV, was retrieved from *M. myotis* and clustered with picornavirus genome sequences detected from *M. ricketti* and *M. schreibersii* bats from China (OR951291.1 Myotis bat parechovirus 2 isolate GX2017, OR951294.1 Myotis bat parechovirus 2 isolate YN2016,) showing sequence identities ranging from 78 to 86.8%. Phylogenetic analysis suggests a close relationship within the *Parechovirus* clade, and its level of sequence identity falls within the International Committee on Taxonomy of Viruses (ICTV) threshold for classification within the genus (< 30% divergence), supporting its identification as a *Parechovirus* member.

The picornavirus from sample F49 (MiSc-F49-PicoV-3), detected in *M. schreibersii*, clustered with picornaviruses from *M. schreibersii* from Spain and Hungary (PP654855.1 Cabezo Gordo bat-associated mischivirus, KP054276.1 BatPV/V12/13/Hun and KP054274.1 BatPV/V8/13/Hun), and a *Miniopterus* species from Algeria (MG888045.1 Algeria/2017/Bejaia), with sequence identities between 86.1% and 90.2%. Based on phylogenetic clustering and sequence similarity and following the ICTV classification criteria for *Picornaviridae* (< 30% divergence), this virus is classified within the genus *Mischivirus*.

Sample F50 from *M. myotis* contained two genomes (MyMy-F50.1-PicoV-3 and MyMy-F50.2-PicoV-3). BLASTn analysis indicated that F50.1 shared a distant sequence similarity (< 78.0%) with picornaviruses previously detected in *M. davidii* and *M. ricketti* from China (OQ716000.1 JM_My.davidii_picorna_1, OQ716001.1 WZ_My.laniger_picorna_2). BLASTn analysis of F50.2 revealed hits with picornaviruses previously detected in *M. myotis* and *M. blythii* from Spain (PP654853 Navarredonda bat picornavirus; PP564854 Almagra bat picornavirus) and *Scotophilus kuhlii* from Vietnam (MZ544240.1 picornavirus isolate 16845 × 77-12), with sequences identities ranging from 79 to 97%. However, phylogenetic analysis revealed that these sequences are divergent from the reference sequences and may represent novel lineages within the *Picornaviridae*.

Similarly, sample F51, also from *M. myotis*, comprised two picornavirus fragments (MyMy-F51.1-PicoV-4 and MyMy-F51.2-PicoV-4). F51.1 shared similarities with picornaviruses obtained from *Scotophilus kuhlii* from China (PP745854 Picornavirales sp. isolate 184_2515), with a percentage identity of 75.1%. BLASTn analysis of sequence F51.2 showed similarities with picornaviruses previously detected in *M. davidii* and *M. ricketti* from China (OQ716000.1 JM_My.davidii_picorna_1, OQ716001.1 WZ_My.laniger_picorna_2), and with *M. myotis* from Romania (JQ916935.1 BtPV/Rcet88-19/Myo_myo/ROU/2008, a classified *Shanbavirus*) with sequence identities ranging from 78.0 to 95.8%. In the absence of an ICTV-defined divergence threshold for this genus, we propose the tentative assignment of F51.2 to *Shanbavirus* based on its phylogenetic placement.

### Partial genome of a novel bat Mastadenovirus

**Fig. 4:**
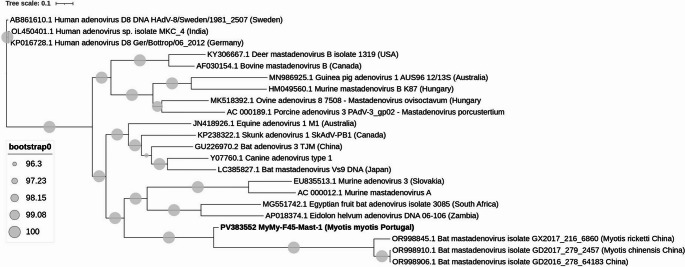
Phylogeny of the *Mastadenovirus* found in this study. The tree was constructed using IQ-TREE2 with the maximum likelihood method based on the GTR + FO model, including 1000 bootstrap replicates. Sequences were aligned with MAFFT. The phylogenetic analysis was performed using the whole genome nucleotide sequences. The tree includes the only *Mastadenovirus*-positive strain identified in this study along with 21 reference strains. Sequence from this study is indicated in bold

A partial *Mastadenovirus* genome sequence of approximately 36,907 nucleotides, representing most of the viral genome, was identified in one bat sample- F45 (MyMy-F45-Mast-1). Phylogenetic analysis placed the adenovirus closely with sequences from *M. chinensis* and *M. ricketti*, all three originating from China (OR998910.1 GD2017_279_2457, OR998906.1 GD2016_278_64183 and OR998845.1 GX2017_216_6860). The percentage sequence identity between our sample and these reference sequences ranged from 84.7 to 88.0%, indicating a moderately high degree of genetic relatedness. The phylogeny of *Mastadenovirus* found in this study can be found in Fig. 4 and phylogenetic analysis was performed on their whole genome nucleotide sequences.

### Dependoparvovirus

We also detected four non-overlapping contigs most closely related to members of the genus *Dependoparvovirus* in one bat sample (F45 – *M. myotis*). Due to the lack of overlap and the limited length of two contigs (< 2 kb, with poor similarity to known sequences), full genome assembly was not feasible. Nevertheless, a phylogenetic analysis was performed using the two longer contigs containing partial capsid gene sequences. The resulting tree, presented in Supplementary Fig. 1, provides preliminary insights into the evolutionary relationships of these sequences. BLASTn analysis of contig MyMy-F45-DepPV-1 showed nucleotide similarities with dependoparvovirus sequences found in *M. ricketti* from China (NC_014468 Bat adeno-associated virus YNM; MH167454.1 Bat adeno-associated virus isolate 1285), with identities around 86%. Contig MyMy-F45-DepPV-2 showed similarities with dependoparvovirus sequences found in *M. ricketti* and *M. chinensis* from China (NC_014468 Bat adeno-associated virus YNM; OR998763.1 Bat adeno-associated virus 2 isolate YN2020), with identities around 80.3%−82.7%.Contig MyMy-F45-DepPV-3 showed highest BLASTn hits with genomes found in *M. ricketti* from China (NC_014468 Bat adeno-associated virus YNM; OR998838.1 Bat adeno-associated virus 2 isolate GD2017_208_21627), with identities around 83%. Contig MyMy-F45-DepPV-4 also demonstrated similarity with genomes found in *M. ricketti* from China (OR998842.1 Bat adeno-associated virus 2 isolate GD2017_211_127613; OR998836.1 Bat adeno-associated virus 2 isolate GD2017_211_127613), with identities around 81%.

### Computational studies of the binding affinity between the Spike protein and APN receptors

The interaction of CoV spike proteins with host cell receptors is a determinant of host specificity and viral entry. In this study, we were able to retrieve the full spike of the CoV genome MiSc-F47-CoV-2. To further explore the potential for cross-species receptor usage, we included porcine APN in our simulations, as the *Pedacovirus* subgenus includes exclusively both bat and pig associated CoVs (Han et al. 2023). Moreover, studies have shown that PEDV and bats likely evolved from a shared ancestor, highlighting the potential role of cross-species transmission between bats and pigs in the evolutionary history of Pedacoviruses (Huang et al. 2013). Additionally, we incorporated human APN to investigate whether this bat CoV could theoretically bind to a human receptor, thereby assessing its zoonotic potential. This approach allows us to evaluate the capacity of bat Pedacoviruses to cross species barriers and informs our understanding of potential transmission routes from bats, through intermediate hosts like pigs, to humans.

**Fig. 5:**
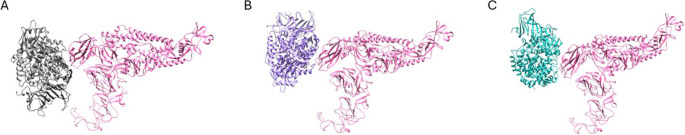
Models of structural interaction between the F47 CoV spike protein (pink) and aminopeptidase N (APN) from (**A**) bat (black), (**B**) porcine (purple), (**C**) human (green)

**Fig. 6:**
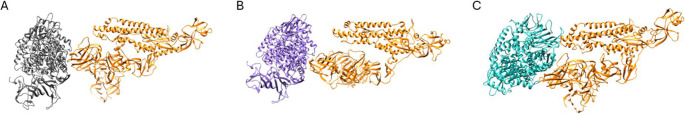
Models of structural interaction between the PEDV spike protein (orange) and aminopeptidase N (APN) from (**A**) bat (black), (**B**) porcine (purple), (**C**) human (green)

In this study, protein docking simulations revealed that the binding of the F47 CoV spike protein was strongest with bat APN and similar with human and porcine APNs (Table 4; Fig. 5). The HADDOCK scores from the protein docking studies between the spike proteins of bat CoV (F47) and the APN from different hosts are presented in Table 4. These values represent the mean and standard deviation of three replicates, indicating the stability and consistency of the docking results. The results show that the interaction between F47 and bat APN yielded a score of −126.1 ± 4.8. For the interaction with porcine APN, the score was − 109.0 ± 4.4, and for human APN, the score was − 106.2 ± 7.6. Figure 5 presents the best structural interaction models obtained from HADDOCK between the RBD of the F47 CoV spike protein and APN from these hosts. The structural differences in these models may reflect variations in binding affinity, as demonstrated by the docking scores. To further explore these findings, docking studies were performed for the PEDV RBD with APN from the same animal species (Table 4; Fig. 6). The interaction between PEDV and bat APN resulted in a score of −90.7 ± 7.5, while with porcine APN, it was − 82.8 ± 2.2, and with human APN, it was − 100.2 ± 5.4.

**Table 4:** HADDOCK scores obtained in the protein Docking studies between the Spike proteins from Bat CoV obtained in this study (F47) and the APN from different hosts, using Haddock 2.4. The values presented correspond to the mean and respective standard deviation of three replicates

	APN		
	**Bat**	**Porcine**	**Human**
F47	−126.1 +/- 4.8	−109.0 +/- 4.4	−106.2 +/- 7.6
PEDV	−90.7 +/- 7.5	−82.8 +/- 2.2	−100.2 +/- 5.4

## DISCUSSION

The intricate relationship between bats and the viruses they harbor has long been a subject of interest, given the unique ecological role of bats as reservoirs for a diverse range of pathogens (Roffler et al. 2024). In Portugal, interactions between bats and humans occur through various channels, including urban roosting in historical buildings, agricultural landscapes where bats contribute to pest control, and ecotourism activities in caves and natural reserves. These interactions can foster awareness and support bat conservation. Yet, a large gap still persists in the characterization of the viral communities present in bats, particularly in Europe, where most studies remain limited in scope.

In this study, we explored the virome of bat populations in Portugal, uncovering significant viral diversity, including coronaviruses (CoVs), picornaviruses, mastadenoviruses (adenoviruses), and dependoparvoviruses. We performed PCR and metagenomic sequencing using the NetoVIR protocol on bat fecal and buccal swab samples collected from Montemor I roost in Portugal. This shelter supports other species of conservation importance, including the Mediterranean horseshoe bat (*Rhinolophus euryale*), greater horseshoe bat (*R. ferrumequinum*), lesser horseshoe bat (*R. hipposideros*), and Mehely’s horseshoe bat (*R. mehelyi*). The surrounding area, characterized by oak savanna-like woodland (locally known as *montados*), which is a type of agroforestry system found in the Iberian Peninsula characterized by open pastures and agricultural land, plays an essential role as a feeding ground (Rainho 2007; Batista 2014).

Through PCR analysis of fecal, buccal, and anal swabs, we detected CoV in three samples (F47, F52 and B51), two from distinct *Myotis* individuals, one from a fecal sample and the other from a buccal swab, and one from *M. Schreibersii*, also from a fecal sample. In contrast, our NGS analysis (which focused on all fecal samples and the buccal sample positive via PCR), identified two CoV-positive results from fecal samples (F45 and F47, two near complete genomes). One of the positive samples (F47) was also positive through PCR, while the other sample (F45) was positive only by NGS and negative by RT-PCR.As shown in Fig. 2, the alignment of primers with the F45 sequence reveals mismatches with Chu-06, and Poon-F primers. These mismatches could explain why the PCR failed to amplify the target, while NGS, with its broader detection capabilities successfully identified the CoVs in the F45 sample.

Previous research highlights that fecal samples often yield the highest detection rates for CoVs, strongly indicating that viral replication predominantly occurs in the gastrointestinal tract (Benkő et al. 2014; Borkenhagen et al. 2019a; Hemnani et al. 2023; Méndez-Rodríguez et al. 2024). Given these findings, we focused our NGS analysis primarily on fecal samples, as they are considered the most likely to contain comprehensive viral genomic data. For the metagenomics, we followed the NetoVIR protocol, an optimized and widely used approach for high-throughput, reproducible sample preparation. This method introduces minimal bias and is particularly suited for fecal samples. (Conceição-Neto et al. 2015b). Additionally, the buccal swab sample that tested positive for CoV by RT-PCR was included to explore any possible alternative sites of replication or shedding. This approach ensured that our sequencing efforts were strategically directed towards maximizing the recovery of relevant viral sequences. The sequencing revealed a wide range of viruses including mammalian, but also insect viruses (data not shown), which is an expected result given the fact that the diet of the sampled Portuguese bats almost consists of insects (Rodrigues et al. 2003; Herrera et al. 2015; Mata et al. 2021). We concentrated exclusively on viruses that can infect mammals, excluding those that target archaea, bacteria, fungi, invertebrates, and plants.

Our study identified two near complete genomes of AlphaCoVs. The clustering of our sequences with CoVs from bats in South Korea (*M. petax*), China (*M. adversus*, *M. chinensis*,* M. fuliginosus* and *M. schreibersii*), and Denmark (*M. dasycneme*) highlights the widespread distribution and genetic diversity of bat-associated coronaviruses. However, this pattern may partly reflect sampling bias, as CoV sequences from Asian bats, particularly from China, are more abundantly represented in public databases compared to those from European or Portuguese bats. The limited availability of regional sequences may contribute to the observed phylogenetic clustering with genetically similar but geographically distant strains. In addition, the migratory behavior of some bat species, may also contribute to the dissemination and genetic mixing of AlphaCoVs across broad geographic regions. Previous studies have documented transboundary bat movements within Europe (Rodrigues and Palmeirim 2008), supporting the possibility of viral exchange along migratory routes. Geographic gaps also exist, with certain regions in Europe lacking comprehensive surveys for bat-associated CoVs, potentially overlooking important reservoirs (Hemnani et al. 2024). These findings show that Portuguese bats may contribute to the broader circulation of AlphaCoVs, reinforcing their role as crucial reservoirs for this viral group.

We also detected picornavirus in several samples, revealing a high degree of sequence divergence both from each other and from known picornavirus sequences in databases. The order *Picornavirales* encompasses nine families, one of which is the family *Picornaviridae*. The identified picornaviruses displayed clustering patterns similar to picornavirus strains found in bat species from China and Europe, particularly within the *Myotis* and *Miniopterus* genera. The identification of six picornavirus genomes across four samples highlights the presence of diverse picornaviruses in Portuguese bats. The clustering patterns suggest a complex evolutionary history, with some strains showing higher similarity to viruses from geographically close regions (e.g., Spain, Hungary and Algeria), while others are more closely related to strains from distant locations such as China. This likely reflects significant gaps in current knowledge and sampling of bat-associated picornaviruses, which may result in unexpected phylogenetic relationships between viruses from distant geographic locations. Additionally, the observed genetic diversity suggests ongoing viral evolution within these bat species (Kramer and Tavakoli 2021; Jones et al. 2023).

Adenoviruses are medium-sized, non-enveloped viruses belonging to the family *Adenoviridae*. The family is divided into two genera: *Mastadenovirus*, which includes viruses infecting mammals (Fitzgerald et al. 2020), and *Aviadenovirus*, comprising those that infect birds (Athukorala et al. 2022).The phylogenetic placement of the detected *Mastadenovirus* alongside strains from *Myotis chinensis* and *Myotis ricketti* in China highlights unexpected relationships between viruses from distant regions. Rather than indicating direct viral exchange or contact between bat populations, this pattern likely reflects significant gaps in the global sampling of bat-associated adenoviruses. Such phylogenetic alignments may arise due to underrepresentation of viral sequences from intermediate regions, emphasizing the need for broader surveillance efforts. Moreover, the ability of adenoviruses to infect multiple bat species and cross species barriers is well-documented within the *Adenoviridae* family (Benkő et al. 2014; Borkenhagen et al. 2019a; Méndez-Rodríguez et al. 2024).

The exact mechanisms that allow the transmission of adenoviruses between species remain poorly understood. While some studies suggest that indirect environmental exposure, such as through contaminated water sources, may play a role in facilitating viral exchange among wildlife, domestic animals, and humans (Borkenhagen et al. 2019b; Simonin 2023), these assumptions are largely based on general patterns of pathogen spread rather than adenovirus-specific evidence. Therefore, the intricacies of cross-species transmission and the ecological or behavioral factors that might enable such events require further investigation in the context of adenoviruses.

*Dependoparvovirus* is a genus within the family *Parvoviridae*. These are parvoviruses that typically require co-infection with a helper virus, such as an adenovirus or a herpesvirus, to complete their replication cycle (Du et al. 2022). This dependency on a helper virus is what gives them their name, *Dependoparvovirus* (Hildebrandt et al. 2020). In this study, *Dependoparvovirus* was detected in *M. myotis* (F45), the same individual in which adenovirus was also identified. The virus found in this study shared high sequence identity with strains from *Myotis* bats in China, with sequence identities ranging from 81.6 to 86.2%. These similarities likely reflect the limited availability of *Dependoparvovirus* sequences globally, rather than direct viral circulation, underscoring the need for broader sampling to better understand their diversity and evolutionary relationships.

To evaluate the risk of cross-species transmission of the bat CoV sequenced in this study, protein docking studies were conducted to analyze the binding affinity between the bat CoV spike RBD and bat, porcine and human APNs. These interactions are critical for understanding host specificity and the potential for spillover to livestock or zoonotic transmission to humans. By evaluating the binding affinities, we gained insights into the virus’s transmission dynamics and its potential to infect other species.

The APN receptors of pigs and humans were included in the docking analysis to assess the potential interaction between the bat CoV spike protein and host receptors. Porcine APN was used due to its known role as the receptor for PEDV. PEDV was included in the docking analysis as a reference alphacoronavirus known to use porcine APN as its entry receptor. Given the sequence similarity between the bat CoV spike protein and PEDV, as well as their shared placement within the *Pedacovirus* subgenus, this analysis aimed to explore whether the bat virus might use a similar entry mechanism. By comparing the binding interactions of the bat CoV spike with those of PEDV, we sought to assess potential similarities in receptor usage and gain insights into possible cross-species transmission pathways (Banerjee et al. 2019; Han et al. 2019). Human APN was included to evaluate the potential risk of zoonotic transmission of this virus. The HADDOCK scores from our protein docking studies reveal important insights into the binding affinities between the spike proteins of bat CoV (F47) and PEDV with APN receptors from different hosts (bat, porcine, and human). The results suggest that the F47 CoV spike protein exhibits the strongest binding affinity with bat APN, yielding a score of −126.1 ± 4.8. This high binding affinity could imply that the F47 CoV is well adapted to the bat host, providing insights into the evolutionary history of the virus and its co-evolution with bat species. Interestingly, the binding affinities between the F47 spike protein and the APNs of both humans and pigs are also strong and comparable, with scores of −106.2 ± 7.6 and − 109.0 ± 4.4, respectively.

This finding suggests a potential capacity for cross-species receptor usage, which could have implications for host range and zoonotic spillover risks. The close binding affinities observed for F47 CoV and human or porcine APN suggest a potential inter-species transmission risk. However, it’s important to note that these are computational studies based solely on the spike protein, and the adaptation of other viral proteins has not been considered. Successful viral infection involves more than just spike-APN interactions, as the function of other viral components is also critical for productive infection.

In contrast, the predicted interaction of PEDV is weakest with porcine APN, with a score of −82.8 ± 2.2, which is quite surprising given that pigs are the natural host for PEDV. Interestingly, the PEDV spike protein demonstrated weaker binding to porcine APN than to human and bat APNs, contrary to the established role of porcine APN as its primary receptor. This unexpected result challenges the established understanding of PEDV spike-receptor interactions and raises intriguing questions about the structural or evolutionary factors influencing binding specificity. In fact, recent studies have suggested that APN may not be the cell receptor of PEDV and that PEDV employs both protein receptors and sugar co-receptors for infection. Additionally, while the significant binding affinity of PEDV spike for human APN (score of −100.2 ± 5.4) may suggest some potential for cross-species interactions, the absence of documented human infections indicates that other factors likely restrict a PEDV spillover. This could be important in understanding potential zoonotic risks and provide insights into the determinants of host specificity.

These findings highlight the need for further experimental validation to confirm receptor usage and assess the functional relevance of these interactions. Moreover, comparative studies of receptor-binding dynamics across different CoVs could provide valuable insights into the evolutionary pressures shaping spike protein-receptor interactions and their role in determining host range. The data might suggest that the strong binding of F47 CoV’s spike to bat APN represents a virus-host relationship optimized through evolutionary pressures, while PEDV’s comparatively weaker binding to pig APN could highlight differences in virus-host adaptation strategies. F47 spike’s comparable affinity for human and porcine APNs also suggests a possible evolutionary plasticity, where the virus might more easily adapt to new hosts with similar APN structures. This binding pattern could also indicate that, while F47 is primarily adapted to bats, it might possess an intrinsic ability to bind APN from other species, potentially aiding cross-species spillover events. In contrast, PEDV’s reliance on additional co-receptors for efficient infection in pigs could suggest a more specialized host relationship, despite the weak APN affinity.

The discovery of CoVs and other viruses in bats in Portugal underscores the importance of monitoring and viral surveillance in bat populations. The detection of viral families commonly associated with mammalian hosts, such as CoVs and picornaviruses, suggests that bats may harbor diverse viral populations with the potential for cross-species transmission.

Given the frequent human-wildlife interactions, such as habitat encroachment, heightened surveillance of bat populations is essential for the early detection of emerging viruses that could pose epidemic risks. This study emphasizes the critical role that bats play in the maintenance of viral diversity, highlighting the need for a “One Health” approach that integrates wildlife monitoring with human health initiatives to prevent potential outbreaks.

This study provided a snapshot of the viral diversity in bat populations in Portugal, contributing to the broader understanding of viral ecology and cross-species transmission. The identification of CoVs and other viral families in these bats underscores the importance of continued research and surveillance to mitigate potential public health threats. Furthermore, it highlights the need for more research on the environmental and ecological factors that influence viral transmission within bat populations. Understanding these dynamics, particularly in regions under high anthropogenic pressure, will be essential for developing effective strategies to mitigate zoonotic risks.

## Supplementary Information

Below is the link to the electronic supplementary material.

Supplementary Material 1(DOCX 159 KB)

## Data Availability

No datasets were generated or analysed during the current study.
