# Supplementary material for: Metagenomic analysis of viral diversity in Portuguese bats
Source: Vet Res Commun. 2025 Sep 20;49(6):319. doi: 10.1007/s11259-025-10888-5 (PMC12450224; doi:10.1007/s11259-025-10888-5)
Supplement: Supplementary file 1 — Supplementary Material 1(DOCX 159 KB) [file 11259_2025_10888_MOESM1_ESM.docx]

Genomes characterization

**CORONAVIRUS**

**MyMy-F45-CoV-1**

Length: 27,692

|  | **Begin** | **End** | **Stop Codons** |
| --- | --- | --- | --- |
| **NT** | 234 | 27,935 |  |
| **CDS** | | |  |
| AVU60_gp1 | 1 | 6,663 | 2 |
| AVU60_gp2 | 1 | 1,108 | 0 |
| AVU60_gp3 | 97 | 220 | 1 |
| AVU60_gp4 | 1 | 55 | 0 |
| AVU60_gp5 | 58 | 228 | 1 |
| AVU60_gp6 | 1 | 430 | 1 |
| **PROTEINS** | | |  |
| ORF1ab polyprotein | 1 | 6,663 | 2 |
| Spike glycoprotein | 1 | 1,108 | 0 |
| Hypothetical protein | 97 | 220 | 1 |
| Small envelope protein | 1 | 55 | 0 |
| Membrane glycoprotein | 58 | 228 | 1 |
| Nucleocapsid protein | 1 | 430 | 1 |

**MyMy-F47-CoV-1**

Length: 28,712

|  | **Begin** | **End** | **Stop Codons** |
| --- | --- | --- | --- |
| **NT** | 26 | 28,773 |  |
| **CDS** | | |  |
| ORF1ab_1 | 1 | 6,897 | 2 |
| ORF1ab_2 | 1 | 4,232 | 2 |
| S | 1 | 1,376 | 1 |
| ORF3 | 1 | 223 | 1 |
| E | 1 | 75 | 1 |
| M | 1 | 252 | 1 |
| N | 1 | 423 | 1 |
| ORF7 | 1 | 249 | 1 |
| **PROTEINS** | | |  |
| ORF1ab polyprotein | 1 | 6,897 | 2 |
| ORF1ab polyprotein | 1 | 4,232 | 2 |
| Spike protein | 1 | 1,376 | 1 |
| ORF3 protein | 1 | 223 | 1 |
| Envelope protein | 1 | 75 | 1 |
| Membrane protein | 1 | 252 | 1 |
| Nucleocapsid protein | 1 | 423 | 1 |
| Hypothetical protein | 1 | 249 | 1 |

**PICORNAVIRUS**

**MyMy-F45-PicoV-1**

Length: 7,421

|  | **Begin** | **End** | **Stop Codons** |
| --- | --- | --- | --- |
| **NT** | 1 | 7,066 |  |
| **CDS** | | |  |
| CAU88_gp1 | 1 | 2,208 | 2 |
| **PROTEINS** | | |  |
| Polyprotein | 1 | 2,208 | 2 |

**MyMy-F49-PicoV-1**

Length: 8,557

|  | **Begin** | **End** | **Stop Codons** |
| --- | --- | --- | --- |
| **NT** | 1 | 6,855 |  |
| **CDS** | | |  |
| FK820_gp1 | 1 | 2,285 | 2 |
| **PROTEINS** | | |  |
| Polyprotein | 1 | 2,285 | 2 |

**MyMy-F50.1-PicoV-3**

Length: 8,351

|  | **Begin** | **End** | **Stop Codons** |
| --- | --- | --- | --- |
| **NT** | 1,187 | 8,351 |  |
| **CDS** | | |  |
| UF59_gp1 | 9 | 2,252 | 1 |
| **PROTEINS** | | |  |
| Polyprotein | 9 | 2,252 | 1 |
| L | 9 | 88 | 1 |
| VP4 | 1 | 64 | 1 |
| VP2 | 1 | 264 | 1 |
| VP3 | 1 | 229 | 1 |
| VP1 | 1 | 261 | 1 |
| 2A | 1 | 32 | 1 |
| 2B | 1 | 163 | 1 |
| 2C | 1 | 364 | 1 |
| 3A | 1 | 90 | 1 |
| 3B | 1 | 20 | 1 |
| 3C | 1 | 209 | 1 |
| 3D | 1 | 467 | 1 |

**MyMy-F50.2-PicoV-3**

Length: 7,656

|  | **Begin** | **End** | **Stop Codons** |
| --- | --- | --- | --- |
| **NT** | 57 | 7,747 |  |
| **CDS** | | |  |
| BaPV1p1 | 1 | 2,384 | 1 |
| **PROTEINS** | | |  |
| Polyprotein | 1 | 2,384 | 1 |
| L | 1 | 74 | 1 |
| VP4 | 1 | 65 | 1 |
| VP2 | 1 | 243 | 1 |
| VP3 | 1 | 234 | 1 |
| VP1 | 1 | 296 | 1 |
| 2A | 1 | 228 | 1 |
| 2B | 1 | 221 | 1 |
| 2C | 1 | 120 | 1 |
| 3A | 1 | 113 | 1 |
| 3B | 1 | 20 | 1 |
| 3C | 1 | 209 | 1 |
| 3D | 1 | 467 | 1 |

**MyMy-F51.1-PicoV-4**

Length: 9,293

|  | **Begin** | **End** | **Stop Codons** |
| --- | --- | --- | --- |
| **NT** | 1 | 9,101 |  |
| **CDS** | | |  |
| BaPV1p1 | 1 | 2,385 | 1 |
| **PROTEINS** | | |  |
| Polyprotein | 1 | 2,385 | 1 |

**MyMy-F51.2-PicoV-4**

Length: 8,440

|  | **Begin** | **End** | **Stop Codons** |
| --- | --- | --- | --- |
| **NT** | 1 | 8,096 |  |
| **CDS** | | |  |
| BaPV1p1 | 1 | 2,252 | 1 |
| **PROTEINS** | | |  |
| Polyprotein | 1 | 2,252 | 1 |
| L | 1 | 88 | 0 |
| VP4 | 1 | 64 | 0 |
| VP2 | 1 | 264 | 0 |
| VP3 | 1 | 229 | 0 |
| VP1 | 1 | 261 | 0 |
| 2A | 1 | 32 | 0 |
| 2B | 1 | 163 | 0 |
| 2C | 1 | 364 | 0 |
| 3A | 1 | 90 | 0 |
| 3B | 1 | 20 | 0 |
| 3C | 1 | 209 | 0 |
| 3D | 1 | 467 | 0 |

**ADENOVIRUS**

**MyMy-F45-Mast-1**

Length: 36,907

|  | **Begin** | **End** | **Stop Codons** |
| --- | --- | --- | --- |
| **NT** | 3,516 | 25,113 |  |
| **CDS** | | |  |
| 05 | 1 | 437 | 1 |
| 06 | 4 | 1,144 | 2 |
| 07 | 4 | 611 | 1 |
| 08 | 1 | 397 | 2 |
| 09 | 31 | 417 | 0 |
| 10 | 35 | 479 | 1 |
| 11 | 1 | 33 | 0 |
| 13 | 36 | 71 | 1 |
| 14 | 1 | 110 | 0 |
| 15 | 1 | 908 | 0 |
| 16 | 1 | 207 | 2 |
| 17 | 101 | 438 | 1 |
| 18 | 91 | 641 | 0 |
| 19 | 136 | 169 | 2 |
| 20 | 1 | 221 | 0 |
| 21 | 1 | 3 | 0 |
| **PROTEINS** | | |  |
| IVa2 protein | 1 | 437 | 1 |
| Polyprotein | 4 | 1,144 | 2 |
| pTP | 4 | 611 | 1 |
| 52K | 1 | 397 | 2 |
| IIIa protein precursor | 31 | 417 | 0 |
| III | 35 | 479 | 1 |
| pVII | 1 | 33 | 0 |
| pX | 36 | 71 | 1 |
| pVI | 1 | 110 | 0 |
| Hexon | 1 | 908 | 0 |
| protease | 1 | 207 | 2 |
| 100K | 101 | 438 | 1 |
| 22K | 91 | 641 | 0 |
| pVIII | 136 | 169 | 2 |
| ES 12.5K | 1 | 221 | 0 |

**PARVOVIRUS**

**MyMy-F45-DepPV-1**

Length: 2,359

|  | **Begin** | **End** | **Stop Codons** |
| --- | --- | --- | --- |
| **NT** | 2,018 | 4,286 |  |
| **CDS** | | |  |
| Rep | 607 | 614 | 1 |
| Cap | 1 | 725 | 1 |
| **PROTEINS** | | |  |
| Rep protein | 607 | 614 | 1 |
| Cap protein | 1 | 725 | 1 |

**MyMy-F45-DepPV-2**

Length: 1,120

|  | **Begin** | **End** | **Stop Codons** |
| --- | --- | --- | --- |
| **NT** | 1 | 1,038 |  |
| **CDS** | | |  |
| Rep | 1 | 280 | 0 |
| **PROTEINS** | | |  |
| Rep protein | 1 | 280 | 0 |

**MyMy-F45-DepPV-3**

Length: 3,138

|  | **Begin** | **End** | **Stop Codons** |
| --- | --- | --- | --- |
| **NT** | 962 | 4,109 |  |
| **CDS** | | |  |
| Rep | 255 | 614 | 1 |
| Cap | 1 | 684 | 0 |
| **PROTEINS** | | |  |
| Rep protein | 255 | 614 | 1 |
| Cap protein | 1 | 684 | 0 |

**MyMy-F45-DepPV-4**

Length: 1,128

|  | **Begin** | **End** | **Stop Codons** |
| --- | --- | --- | --- |
| **NT** | 1 | 1,038 |  |
| **CDS** | | |  |
| Rep | 1 | 280 | 0 |
| **PROTEINS** | | |  |
| Rep protein | 1 | 280 | 0 |

**
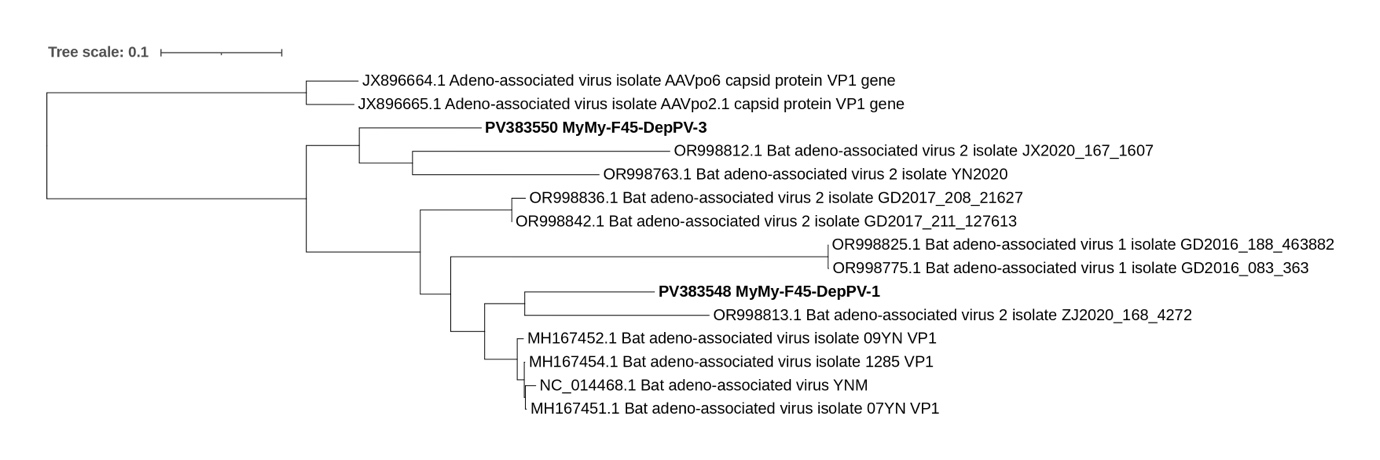
**

Figure 1: Phylogeny of Dependoparvovirus sequences detected in this study. The tree was constructed using the capsid gene sequences of two contigs from sample F45 and the 10 most similar reference sequences identified via BLASTn. Sequences were aligned with MAFFT, and the tree was generated using the PHYML tree builder with 100 bootstrap replicates in Geneious Prime® 2025.1.3. Two additional contigs were excluded from the analysis due to insufficient length (<2 kb) and low similarity to known reference genomes. Sequences from this study are indicated in bold
